# Systematic Characterization of bZIP Transcription Factors Required for Development and Aflatoxin Generation by High-Throughput Gene Knockout in *Aspergillus flavus*

**DOI:** 10.3390/jof8040356

**Published:** 2022-03-30

**Authors:** Qianqian Zhao, Hao Pei, Xiaoling Zhou, Kai Zhao, Min Yu, Guomin Han, Jun Fan, Fang Tao

**Affiliations:** 1 School of Life Sciences, Anhui Agricultural University, Hefei 230036, China; zhaoqianqian202203@163.com (Q.Z.); 19720367@stu.ahau.edu.cn (H.P.); zhouxiaoling@stu.ahau.edu.cn (X.Z.); yumin@stu.ahau.edu.cn (M.Y.); 2 The National Engineering Laboratory of Crop Stress Resistance Breeding, Anhui Agricultural University, Hefei 230036, China; zhaokai@stu.ahau.edu.cn (K.Z.); guominhan@ahau.edu.cn (G.H.); fanjun@ahau.edu.cn (J.F.)

**Keywords:** *Aspergillus flavus*, bZIP transcription factors, mycelial growth, conidiation, sclerotia, aflatoxin, stress response, pathogenicity

## Abstract

The basic leucine zipper (bZIP) is an important transcription factor required for fungal development, nutrient utilization, biosynthesis of secondary metabolites, and defense against various stresses. *Aspergillus flavus* is a major producer of aflatoxin and an opportunistic fungus on a wide range of hosts. However, little is known about the role of most bZIP genes in *A. flavus*. In this study, we developed a high-throughput gene knockout method based on an *Agrobacterium*-mediated transformation system. Gene knockout construction by yeast recombinational cloning and screening of the null mutants by double fluorescence provides an efficient way to construct gene-deleted mutants for this multinucleate fungus. We deleted 15 bZIP genes in *A. flavus*. Twelve of these genes were identified and characterized in this strain for the first time. The phenotypic analysis of these mutants showed that the 15 bZIP genes play a diverse role in mycelial growth (eight genes), conidiation (13 genes), aflatoxin biosynthesis (10 genes), oxidative stress response (11 genes), cell wall stress (five genes), osmotic stress (three genes), acid and alkali stress (four genes), and virulence to kernels (nine genes). Impressively, all 15 genes were involved in the development of sclerotia, and the respective deletion mutants of five of them did not produce sclerotia. Moreover, MetR was involved in this biological process. In addition, *HapX* and *MetR* play important roles in the adaptation to excessive iron and sulfur metabolism, respectively. These studies provide comprehensive insights into the role of bZIP transcription factors in this aflatoxigenic fungus of global significance.

## 1. Introduction

*Aspergillus flavus* is a saprophytic opportunistic fungus that is infamous for its production of the hepatocarcinogenic secondary metabolites known as aflatoxins. These mycotoxins frequently contaminate a wide range of crops, such as maize (*Zea mays* L.), peanut (*Arachis hypogeae* L.) and tree nuts, causing substantial economic losses worldwide. The contamination of food or feed with aflatoxins poses a serious threat and health risk to humans and animals.

*A. flavus* normally reproduces with asexual spores. These conidia are an efficient form of mass dissemination and serve as the primary inocula. Initially, germination of the spores and subsequent vegetative growth forms the mycelia. Some of the hyphal cells stop mycelial growth and begin asexual development by forming conidiophores that bear multiple chains of conidia. In severe environmental conditions, sclerotia are formed by fusion and aggregation of the mycelia and can remain dormant for long periods of time until favorable conditions allow for germination and the production of conidia. The fungus can also reproduce sexually. Unlike *A. nidulans*, which produces meiospores (i.e., ascospores) in the sexual fruiting bodies known as cleistothecia, the sexual ascospores of *A. flavus* are found within ascocarps present in the matrix of stromata [1,2]. If the environmental conditions are favorable, mycelia, conidia and sclerotia can produce aflatoxin.

Transcription factors (TFs) are essential regulators of gene expression in eukaryotic cells and play a major role in fungal development, pathogenesis and responses to the environment [3]. The Fungal Transcription Factor Database (http://ftfd.snu.ac.kr, accessed on 6 January 2020) contains 118,563 putative fungal TFs classified into 61 families in 249 fungal and six oomycete species. In *A. flavus*, 647 putative TFs corresponding to 5.13% of 12,604 genes were identified in the genome (www.ftfd.snu.ac.kr, accessed on 6 January 2020). In addition, several TFs that modulate the development and secondary metabolism of *A. flavus* have been reported. The Zn2Cys6 TF, AflR, regulates aflatoxin biosynthesis by binding to the palindromic sequence 5′-TCGN5CGA-3′ in the aflatoxin pathway cluster gene promoters [4,5,6]. A homeobox TF, Hbx1, is involved in development and the production of aflatoxin [7,8]. The Far TFs, FarA and FarB, are involved in various aspects of fatty acid metabolism [9]. A C2H2 TF, RsrA, that regulates stress responses is required for both meiotic and mitotic spore development and affects the production of spores and sclerotia [10]. Another C2H2 TF, mtfA, governs the production of aflatoxin and the normal maturation of sclerotia and increases the pathogenicity of *A. flavus* [11].

The basic leucine zipper (bZIP) transcription factor family is one of the largest and most diverse TF families in fungi. The bZIP TFs are defined by a conserved basic region responsible for DNA-binding, followed by a leucine zipper that forms a homo- and hetero-dimerization interface between the bZIPs. In recent years, fungal genome sequencing has provided a platform for the systematic analysis of the primordial bZIPs based on conserved domains. Members of bZIPs in some species have been thoroughly investigated on the genomic level. For example, 14 members of the bZIP TF family are present in *Saccharomyces cerevisiae*, nine in *Neurospora crassa* [12], 22 in *Magnaporthe oryzae* [13] and 22 in *Fusarium graminearum* [14]. In addition, 26 bZIP genes in *Coniothyrium chrysosperma* [15], 34 in *Coniothyrium minitans* [16], 28 in *Ustilaginoidea virens* [17], and 38 in *Phytophthora infestans* [18] were also identified at the genomic level.

The bZIP TFs are involved in many critical processes, such as development, the utilization of nutrients, biosynthesis of secondary metabolites, and defense against various stresses. One bZIP member designated FlbB is involved in the transcriptional activation and developmental progression of *brlA* in *A. nidulans* [19,20,21]. In *A. fumigatus*, it is required for gliotoxin production beyond asexual development [22]. Other members, such as CpcA and JlbA, are TFs that respond to amino acid starvation in *A. nidulans* [23] and *A. fumigatus* [24]. Another member, HacA, mediates the response to unfolded proteins, which involves a complex signal pathway related to the folding, quality control and transport of secreted proteins in species of *Aspergillus* [25,26,27] and *Trichophyton rubrum* [28]. HapX is indispensable for the adaption to iron starvation and crucial for virulence in many fungi, such as *A. fumigatus* [29,30], *A. nidulans* [31], *F. graminearum* [32], *F. oxysporum* [33], *Verticillium dahliae* [34] and *Beauveria bassiana* [35]. In addition, MetR contributes to the regulation of sulfur and methionine metabolism [36,37,38,39], while MeaB affects the repression of nitrogen metabolites [40,41,42]. Moreover, some bZIP TFs mediate the oxidative stress response and play important roles in fungal development, particularly secondary metabolism. Nap1 in *A. nidulans*, an ortholog of yeast Yap1 [43], regulates sexual development and affects the response to oxidative stress and the biosynthesis of mycotoxin sterigmatocystin (ST) [44,45]. Apyap1 in *A. parasiticus* [46] and Aoyap1 in *A. ochraceus* [47] have also been verified to be involved in antioxidant defenses and affect the biosynthesis of mycotoxins. In addition, RsmA (Yap-like bZIP) regulates gliotoxin cluster metabolites in *A. fumigatus* [48] and ST production in *A. nidulans* [49]. Although a few bZIPs have been characterized in *A. flavus*, such as MeaB [40], Afap1 [50] and AflRsmA [51], the function and regulation of most bZIPs produced by *A. flavus* remain to be elucidated.

In this study, we identified and characterized the bZIP genes at the genome level in *A. flavus*. In the 17 bZIP genes predicted, except for *MeaB*, *Afap1* and *Fcr3* (*AflRsmA* ortholog), which have been reported, the functions of 14 bZIP genes have not been verified in this fungus. We used a high-throughput gene knockout method based on an *Agrobacterium*-mediated transformation system to delete these bZIP genes. In our method, gene-deleted cassettes were constructed by yeast recombinational cloning, and the null mutants were identified by a double fluorescence and negative (target gene) screening system. We used this system to generate 15 bZIP genes in null mutants with homogeneous nuclei (HMN) and studied the involvement of these genes in mycelial growth, conidiation, sclerotial development, aflatoxin production, abiotic stress, and virulence on kernels. Our work will help to understand the regulation of the bZIP transcription factor family in development, secondary metabolism, oxidative stress response and pathogenicity of *A. flavus* and provide an efficient method to construct gene-deleted mutants at the genome level in this multinucleate fungus.

## 2. Materials and Methods

### 2.1. Strains and Culture Conditions

*Escherichia coli* strain DH5α and *Agrobacterium tumefaciens* strain AGL-1 were grown in DYT media (tryptone, 16 g/L; yeast extract, 10 g/L; and NaCl, 5 g/L; with 15 g/L agar added to prepare the plates) at 37 and 28 °C, respectively.

*Saccharomyces cerevisiae* strain FY834 (*MATa*; *his*Δ*200*; *ura3-52*; *leu2*Δ*1*; and *lys2*Δ*202*) was refreshed on YPD agar medium (yeast extract, 10 g/L; glucose, 20 g/L; and peptone, 20 g/L) at 28 °C for 48 h, and then used to prepare competent cells with the PEG/LiAC method [52]. Yeast transformants were selected on Sc-U medium (yeast nitrogen base, 1.7 g/L; ammonium sulfate, 5 g/L; casein hydrolysate, 5 g/L; adenine hemisulfate salt, 20 mg/L; and glucose, 20 g/L).

The *A. flavus* wild-type isolate NRRL 3357 [53] was used as the recipient strain for fungal genetic transformation. The isolate was grown at 30 °C on potato dextrose agar (PDA) (Difco Laboratories, Inc., Detroit, MI, USA) plates in the dark for 7 days. Fresh conidia were then harvested and used for the transformation experiments. Wickerham medium (WKM) was used to observe the formation of sclerotia [54]. The analysis for aflatoxin was conducted on strains grown on YES media (20 g/L yeast extract, 150 g/L sucrose, and 15 g/L agar).

### 2.2. Prediction of bZIPs in A. flavus

Previously reported bZIP proteins of *A. flavus* [40,50,51] and Hidden Markov Model (HMM) profiles of the bZIP proteins were aligned against the *A. flavus* NRRL 3357 proteins (http://fungi.ensembl.org, v2.0, accessed on 8 January 2020) using BLASTP. The candidate genes were verified in the Pfam database (http://pfam.xfam.org/, accessed on 15 January 2020) and SMART (http://fungi.ensembl.org, v2.0, accessed on 15 January 2020). Conserved motifs among the bZIP genes were examined using MEME software (Multiple Expectation Maximization for Motif Elicitation).

### 2.3. Generation of the Yeast–Escherichia–Agrobacterium Shuttle Vector pUM-GFP

To construct the pUM-GFP vector, a promoter fragment of the *A. flavus tef1* gene (838 bp) and *gfp* gene (720 bp) were amplified together from the pFC-eGFP vector [53] with primers Ptef1-up/Pgfp-down (Table S1), and inserted into the *Xho* I/*Bam*H I sites of the pUM vector [53].

### 2.4. High-Throughput Construction of the Gene Knockout Vector

Gene-deletion cassettes were constructed using a yeast in vivo homologous recombination system. It contained a 900–1200 bp DNA fragment of the 5′ and 3′ flanking sequences of the target gene and the *Ble*-*RFP* (BR) expression cassette. Flanking sequences of the 17 bZIP genes were retrieved from the *A. flavus* NRRL 3357 genome. Primers for specific flank sequences of the target genes were designed with primer premier 5.0 as shown in Table S1. For each gene, primers 5f/5r and 3f/3r were designed and synthesized with the common 30-nt 5′ homologous regions:

5f: GGCATGGACGAGCTGTACAAGTAAGGATCC… (homologous to pUM-GFP)

5r: CAATAAGGAGCTTACTCCTCCTTGACACCA… (homologous to the BR cassette)

3f: GTAAGCGCCCACTCCACATCTCCACTCGAC… (homologous to the BR cassette)

3r: TAAACGCTCTTTTCTCTTAGGTTTACCCGC… (homologous to pUM-GFP)

The BR cassette was constructed by the substitution of GFP gene in the pDHBG vector [55] with RFP gene to generate the pDHBR vector.

The flank fragments of target genes were produced from the genomic DNA of *A. flavus* NRRL 3357. The BR cassette fragment was amplified with the primers Pbr-f/ Pbr-r from pDHBR. All the PCR products were verified by sequencing. The flank fragments of the target gene, the BR cassette fragment and pUM-GFP that had been linearized by *Bam*H I/*Hin*d III were mixed and transformed into FY834 competent cells following a small-scale yeast transformation according to the manufacturer’s instructions for pYES2 (Invitrogen, Carlsbad, CA, USA) and selected on Sc-U media. The homologous recombination plasmid products were purified using a TIANprep Yeast Plasmid DNA Kit (DP112; Tiangen Biotech Co., Ltd., Beijing, China) and then transformed into *E. coli* DH5α competent cells. The DNA sequence of the final assembled plasmid designated pKO-x (x represents the target gene) was confirmed by PCR and DNA sequencing, after which it was transformed into the AGL-1 strain. The primers used in this study are shown in Table S1.

### 2.5. Generation of the Knockout Mutants by ATMT

pKO-x plasmids that harbored gene-deletion cassettes were transformed into *A. flavus* using the *Agrobacterium tumefaciens*-mediated transformation (ATMT) method [55]. Simply, the mixture of *A. flavus* conidial suspensions and *A. tumefaciens* cultures was cultured on cellulose nitrate membranes placed on co-cultivation media at 22 °C for 2 days and then transferred to selective media that contained 300 μg/mL cefotaxime, 60 μg/mL streptomycin and 100 μg/mL zeocin and incubated at 28 °C in the dark until colonies appeared. The individual colonies were transferred to new selection media and grown at 28 °C for 3–4 days.

### 2.6. Identification of Gene-Deleted Mutants by Double Fluorescence

The expression of GFP and RFP in the *A. flavus* transformants was analyzed using a Leica DM5000 B fluorescence microscope (Leica, Wetzlar, Germany). Selected transformants were incubated on PDA plates at 30 °C for 2–5 days, and then spores, mycelia or conidiophores were collected for fluorescence analysis. The ectopic transformants emitted both green and red fluorescence; putative null mutants only emitted red fluorescence, and the wild-type strain NRRL 3357 did not respond when excited under the fluorescence microscope. The transformants with red fluorescence were picked out and inoculated on a new selective medium to isolate single spores. Each isolate was studied further under the fluorescence microscope.

### 2.7. Verification of Gene-Deleted Mutants by PCR and Southern Blotting

The genomic DNA was extracted using an amended CTAB method [55]. The putative null mutants with red fluorescence were identified by negative screening double PCR as previously described [56]. PCR was performed using the primers Pnull_f/Pnull_r internal to the target gene (Table S1B) and the primers Ptub-f/Ptub-r for the *β-tubulin* gene. The PCR reaction system was as follows: 1.0 μL Px-f/Px-r (10 μM), 0.3 μL Ptub-f/Ptub-r (10 μM), 2.5 μL 10 × PCR buffer, 0.4 μL dNTP mix (25 μM), 0.3 μL Taq (5U/μL), 19.5 μL ddH_2_O and 1 μL genomic DNA. The amplification reaction was carried out at 94 °C for 2 min, 32 cycles of 94 °C for 30 s, 58 °C for 45 s and 72 °C for 30 s, followed by 72 °C for 5 min. If the target gene was deleted, there was only one band for *β-tubulin* with 580 bp in homogeneous nuclei (HMN) strains. Otherwise, there were two bands in a heterogeneous nuclei (HTN) strain, one for *β-tubulin* and another for the target gene.

The null mutants were also identified by positive PCR. One primer P1 or P4 was limited in the genomic DNA outside of the 5′ or 3′ flanking fragment in gene-deletion cassettes, and another primer P2 or P3 was limited in the BR cassettes. In this study, only P1/P2 primers were used (Table S1C).

For Southern blotting, DNA hybridization probes were amplified with primers (Table S1D) and labeled with digoxigenin-dUTP using DIG-high prime according to the manufacturer’s instructions (11585614910; Roche, Shanghai, China). The Southern blots were performed as previously described [53].

One deletion mutant was selected for each bZIP gene and used in the phenotypic characterization.

### 2.8. Complementation of Null Mutants with Native Genes

The mutant Δ*MetR* was complemented with native gene copies from the wild-type strain NRRL 3357 using a site-specific integration system [53]. Briefly, the fragments that contained the native promoter region of the gene, full-length coding region and terminator sequences were amplified from NRRL 3357 genomic DNA with the primers PMRcom-f/PMRcom-r (Table S1D) and then cloned into the pUM vector using the yeast gap repair approach to generate the pFC-MetR vector. The sequenced complementary plasmids were transformed into the mutants using the ATMT method. The spores of Δ*MetR* harvested from PDA supplemented with 5 mM L-methionine were used as the transforming receptor. The transformants were screened on MM media supplemented with 150 μg/mL carboxin. The gene-rescued transformants were validated by quantitative PCR (qPCR).

### 2.9. RNA Isolation and Quantitative PCR

To investigate the transcriptional inhibition of aflatoxin biosynthesis, conidial suspension (3 × 10^4^ spores) was seeded onto YES plates and incubated at 28 °C. The mycelia of *A. flavus* grown for three days were collected for total RNA isolation using the RNAiso Plus reagent (TaKaRa Co., Ltd., Otsu, Shiga, Japan) according to the manufacturer’s instructions. cDNA was synthesized from 1 µL of total RNA by reverse transcription using a TransScript One-Step gDNA Removal and cDNA Synthesis SuperMix Kit (Transgen Biotech Co. Ltd., Beijing, China). Reverse transcription (RT) was performed by incubating the mixture for 5 min at 65 °C, and the PCR program was as follows: 25 °C for 10 min, 42 °C for 15 min, 85 °C for 5 s and 40 °C for 5 s.

*aflR* and *aflS*, the regulatory genes of the aflatoxin biosynthetic pathway, were selected for quantitative analysis. qPCR (PikoReal 96 Real-Time PCR System; Ventaa, Finland) was conducted using the TB Green^®^ Premix Ex TaqTM II (TaKaRa Co., Ltd.), in a final volume of 20 µL, consisting of 10 µL TB Green Premix Ex Taq II (2×), 0.5 µL of each primer (10 µM) and 1 µL cDNA. The qPCR program included an initial denaturation at 95 °C for 30 s, followed by a 2-step PCR, 40 cycles of 95 °C for 5 s and 60 °C for 30 s. The *β-tubulin* gene was used as the reference gene, with three biological replicates assessed for each sample. The relative levels of expression were calculated using the comparative CT (2^−∆∆CT^) method.

### 2.10. Fungal Growth, Conidial and Sclerotial Production

To investigate the development of all the mutants, fresh spores were harvested from 7-day-old PDA plates with 0.01% Triton X-100 and diluted with sterilized water to a concentration of 10^6^ spores/mL after filtration through lens wiping paper to remove hyphae. The spores of Δ*MetR* harvested from PDA supplemented with 5 mM L-methionine. The spore count was estimated using a hemocytometer. A 10 μL aliquot of the spore suspension was used as inoculum for all the cultivation states. The wild-type strain NRRL 3357 was used as the control, and three replications were conducted for each test.

To determine the fungal growth, a spore suspension was inoculated onto fresh MM and PDA media. Cultures were grown at 30 °C for 7 days. The diameter of the mycelial colony was recorded, and the colony images were photographed at 7 days post inoculation. To quantitatively compare the production of conidia, they all were washed off from a 7-day-old culture using a solution of 0.01% Triton X-100 and counted in a hemocytometer.

The sclerotia were analyzed by centrally seeding a spore suspension onto the WKM plates and incubating them in the dark for 10 days at 30 °C. The conidia were then washed off the plates with 75% alcohol, and the remaining sclerotia were counted under a microscope.

### 2.11. Abiotic Stress Conditions

For oxidative stress, a 10 μL spore suspension (1 × 10^6^ spore/mL) of *A. flavus* wild-type and bZIP mutants was point inoculated onto fresh PDA media supplemented with 3, 6 and 8 mM H_2_O_2_, respectively. PDA medium supplemented with 1.5 M sorbitol was used to assess osmotic stress, while PDA medium supplemented with 400 μg/mL CFW was used to assess cell wall stress. pH 5.0 and pH 9.0 MM media were used for acid and alkali stress, respectively. After 3 days of culture in darkness at 30 °C, the diameters of the mycelial colonies were recorded. Three replicates were analyzed for each stress. The growth inhibition rate of each mutant was calculated as follows:

Growth inhibition rate (%) = (colony diameter under no stress conditions − colony diameter under stress conditions)/colony diameter under no stress conditions × 100.

### 2.12. Aflatoxin Analysis

The production of AFB1 was quantitatively compared as previously described [57]. The deleted mutants cultivated on YES agar were used to analyze the toxins. The plate was overlaid with sterile cellophane sheets and then centrally single-point inoculated with a 10 μL spore suspension (1 × 10^6^ spore/mL). The wild-type fungus was used as the positive control. After 4 days of incubation at 28 °C, the fungal biomass was scraped from the plates and weighed, and extracted in a 50 mL tube by incubation with 5 mL of methanol at room temperature with shaking at 200 rpm for 2 h. The supernatant was then collected by centrifugation at 3000× *g* for 10 min at room temperature and filtered through a syringe filter (0.22 µm, Alltech, Nicholasville, KY, USA). Each sample was analyzed by a Waters 600 Controller HPLC equipped with a fluorescence detector (Waters 2475 Multi λ Fluorescence Detector; Milford, MA, USA). The chromatogram was recorded at 365 nm excitation and 465 nm emission wavelength using a reverse-phase column Luna 3u C18 (2), 150 mm × 4.6 mm × 3 µm (Phenomenex, Torrance, CA, USA), and an isocratic mobile phase with a flow rate of 0.6 mL min^−1^ that consisted of a mixture of methanol:water (55:45). Three replicates were analyzed for each concentration. AFB1 production was measured as μg/g of mycelia.

### 2.13. Kernel Infection Assay

A laboratory kernel infection assay (KIA) was performed as previously described with modifications [57]. Conidia of the *A. flavus* strains were harvested from the PDA plates using a solution of 0.01% Triton X-100 and adjusted to a cell density of 2 × 10^6^ /mL. Undamaged maize kernels were sterilized with 75% ethanol and 1% NaClO for 5 min in turn and dipped into conidial suspension for 5 min. The kernels were then placed in 35 mm Petri dishes without a lid, and these small dishes were then placed in a large Petri dish (90 × 20 mm) with the embryo up and incubated at 30 °C for 7 days. High humidity (>95% relative humidity (RH)) was maintained by adding double-distilled water to the large dishes. An untreated sample served as the control, and three replications were conducted for each test. Infection was designated as visible mycelia and conidia on the surface of the kernel. The rate of infection was calculated by dividing the infected area by kernel surface area. Spores were also harvested and counted with a hemacytometer.

### 2.14. Statistical Analysis

All experimental results were reported as mean ± standard deviation (SD). Statistical analyses were performed using GraphPad Prism 8.0 software (GraphPad Software, San Diego, CA, USA). A Dunnett test was used to determine the difference between each bZIP mutant and wild-type. The significance level was set at *p* < 0.05.

## 3. Results

### 3.1. Identification of bZIP Transcription Factors in the Aspergillus flavus Genome

**Figure 1:**
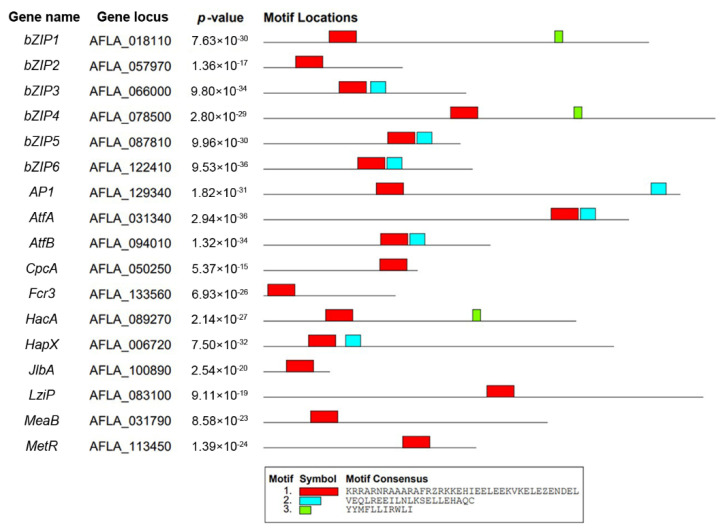
Analysis of the conserved motifs of bZIP family transcription factors in *Aspergillus flavus*.

Seventeen putative bZIP genes were identified in the *A. flavus* NRRL 3357 genome (http://fungi.ensembl.org, v2.0, accessed on 15 January 2020). Except for *bZIP1* to *bZIP6* designated in this paper, 11 bZIPs had been annotated in GenBank. Among those, the functions of *AP1* and *MeaB* have been experimentally verified. Conserved motifs of the bZIP proteins were identified using the MEME software suite and showed that all the proteins contained at least one bZIP domain, which is shown in red in Figure 1 (*p* < 0.001). In addition, seven members of the bZIP proteins also contain adjoining leucine-rich motifs (shaded blue).

### 3.2. Strategy of the Double Fluorescence Knockout System in A. flavus

**Figure 2:**
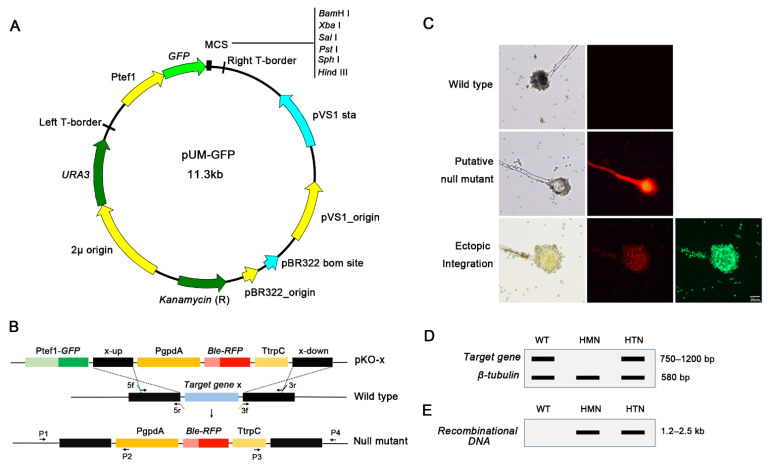
Gene knockout strategy in *Aspergillus flavus*. (**A**) Features of the yeast–*Escherichia*–*Agrobacterium* shuttle vector pUM-GFP. (**B**) Construction of the double fluorescence knockout vector pKO-x and deletion of the targeted gene (x) by homologous recombination in the fungi. (**C**) The transformants were screened by double fluorescence. Putative mutants have RFP fluorescence; ectopic transformants have both GFP and RFP fluorescence, and the wild-type lacks fluorescence. Bar = 25 μm. (**D**) Negative double PCR to identify the null mutants with homogeneous nuclei (HMN) using the *β-tubulin* gene as a positive control. (**E**) HMN mutants were verified by positive PCR for a unique recombinational DNA fragment. GFP, green fluorescent protein; HMN, homogeneous nuclei; HTN, heterogeneous nuclei; RFP, red fluorescent protein; WT, wild-type.

To quickly construct gene-deletion cassettes and efficiently identify null mutants from the numerous transformants, we developed a high-throughput gene knockout system using a yeast–*Escherichia*–*Agrobacterium* shuttle vector, pUM-GFP. This vector contains the *URA3-2μ* origin sequence from the yeast plasmid pYES2 and a GFP reporter gene under the control of the *A. flavus* tef1 promoter. The yeast replicon design makes it highly convenient and efficient to construct multiple gene-deletion cassettes by yeast recombinational cloning, regardless of the potential restriction sites in the sequences. The 5′ and 3′ flanking fragments of the targeted gene (x), designated x-up and x-down, the *Ble-RFP* (BR) fusion expression cassette and the linearized pUM-GFP vector were transformed to yeast for one-step in vivo recombination. The final pKO-x vector contained two fluorescence reporter genes, *GFP* and *RFP* (Figure 2A,B). The gene-deletion cassettes in the pKO-x vector were transformed to the wild-type fungus using the ATMT method. The transformants were grown on positive selection plates and were then identified by double fluorescence screening. The transformants that emitted only red fluorescent protein (RFP) fluorescence were identified as putative null mutants; the ones that emitted both RFP and green fluorescent protein (GFP) fluorescence were ectopic insertional transformants, and the ones that did not fluoresce were the wild-type (Figure 2C).

The putative null mutants were further identified to have homogeneous nuclei in their conidia by negative double PCR of the target and *β-tubulin* genes. Theoretically, the putative null mutants emit only RFP, and a lack of GFP fluorescence suggested that the target gene had been recombinationally replaced by the *Ble-RFP* cassette. In addition, only one band for *β-tubulin* could be amplified in negative PCR. However, most of the conidia of *A. flavus* are multinucleate. In rare cases, a few putative null mutants harbored heterogeneous nuclei (HTN), a condition in which wild-type and recombinational nuclei coexisted in one strain. Thus, another band for the target gene could be amplified from the HTN mutants. Through negative PCR, the null mutants with homogeneous nuclei (HMN) were identified, and only one band for a *β-tubulin* gene of 580 bp could be amplified in this mutant (Figure 2D). HMN mutants were then verified by positive PCR of the gene-deletion cassettes. In positive PCR, one primer was limited in the genomic DNA outside of the x-up or x-down, while another primer was limited in the BR cassette. One band of approximately 1.2–2.5 kb in length was amplified from the HMN mutants (Figure 2E), which suggested that the foreign fragment (BR cassette) had replaced the target gene. Although the same band could be amplified from the HTN mutants, the interference would be eliminated by negative PCR.

### 3.3. Construction of bZIP Deletion Mutants in A. flavus

The 17 bZIP genes that were predicted to contain the two verified genes were all selected to generate gene-deletion mutants using the double fluorescence knockout system. As a result, 201 resistant transformants for all bZIP genes were obtained. A total of 96 only had red fluorescence and 57 had double fluorescence, while 48 lacked fluorescence. Further, double PCR and positive PCR (Figure S1A) for the transformants showing only the red fluorescence allowed for the selection of 61 HMN mutants, while the other 35 were HTN mutants (Table S2). The knockout event was also verified by a Southern blot assay of two mutants (Figure S1B). The 61 HMN mutants are members of the 15 bZIP genes. The knockout rate of 15 genes ranged from 6.25% (*LziP*) to 100% (*JlbA*) (Table S2). However, *bZIP3* and *HacA* were only obtained in HTN mutants. The causes may lie in the following: (1) The genes could be involved in fungal nutrient metabolism. Therefore, their deletion may have resulted in an inability of the mutant to grow on minimal medium (MM) selection media. (2) The genes may be essential. The homozygous mutant is lethal. In these cases, a heterozygote with heterogeneous nuclei could grow.

### 3.4. Phenotypic Analyses of the bZIP Transcription Factor Deletion Mutants

**Figure 3:**
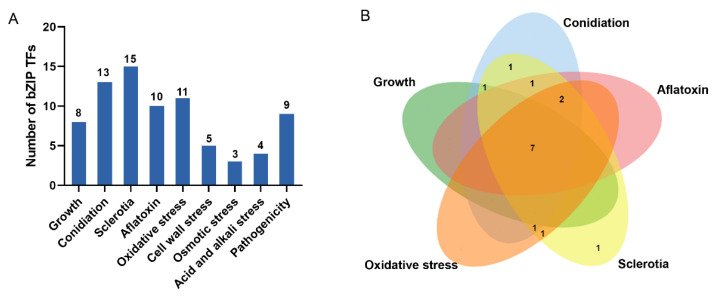
Phenotypic analysis of 15 bZIP transcription factor mutants in different biological processes of *Aspergillus flavus*. (**A**) Number of bZIPs that display mutant phenotypes in different biological processes. (**B**) A Venn diagram that depicts the number of mutant phenotypes. The phenotypes included developmental characteristics (mycelial growth, conidiation, and sclerotial production), aflatoxin and oxidative stress.

The phenotypes of HMN mutants of 15 bZIPs were analyzed at different developmental stages, including developmental characteristics, such as mycelial growth, conidiation, and sclerotial production. The production of aflatoxin B1 (AFB1), response to stress and virulence to kernels were also studied. The results showed that eight TF genes were involved in mycelial growth, 13 genes in conidial production, 15 genes in sclerotial production, and 10 genes were involved in the biosynthesis of aflatoxin. Eleven TF genes were involved in H_2_O_2_ stress, five in cell wall stress, three in osmotic stress, and four in acid and alkali stress. Nine TF genes were involved in virulence to kernels (Figure 3A, Table 1 and Table S3). Each TF gene was involved in multiple biological processes. There were seven TF genes that were simultaneously involved in growth, conidiation, sclerotial and aflatoxin production and oxidative stress response (Figure 3B). In addition, *MetR* was involved in all the processes examined (Table 1).

### 3.5. bZIP Transcription Factors Involved in Fungal Growth

**Figure 4:**
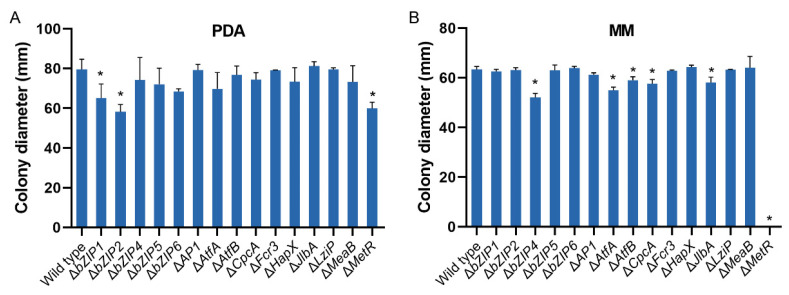
Mycelial growth of *Aspergillus flavus* strains on media. In total, 1 × 10^4^ spores were cultured on PDA (**A**) and MM (**B**) media for 7 days at 30 °C. The diameter of colonies was measured. Error bars represent the SD. * *p* < 0.05, significant difference from the wild-type group as estimated by a Dunnett test. MM, minimal media; PDA, potato dextrose agar; SD, standard deviation.

The fungal growth of the null mutants with HMN of the 15 bZIP transcription factors was studied on PDA and MM media. Figure S2 shows the colony phenotype of each mutant and the control strain. The mycelia of eight of these null mutants differed significantly compared with those of the wild-type fungus (Figure 4, Table S3). In detail, Δ*bZIP1* and Δ*bZIP2* only had smaller colonies on PDA at 82% and 73.2%, respectively. The growth of colonies of five bZIPs mutants (Δ*bZIP4*, Δ*AtfA*, Δ*AtfB*, Δ*CpcA* and Δ*JlbA*) was only reduced on MM. Δ*MetR* reduced growth on PDA at 24.7% and did not grow on MM. Moreover, Δ*LziP* exhibited a “fluffy” phenotype on MM (Figure S2).

### 3.6. bZIP Transcription Factors Involved in Conidial Production

**Figure 5:**
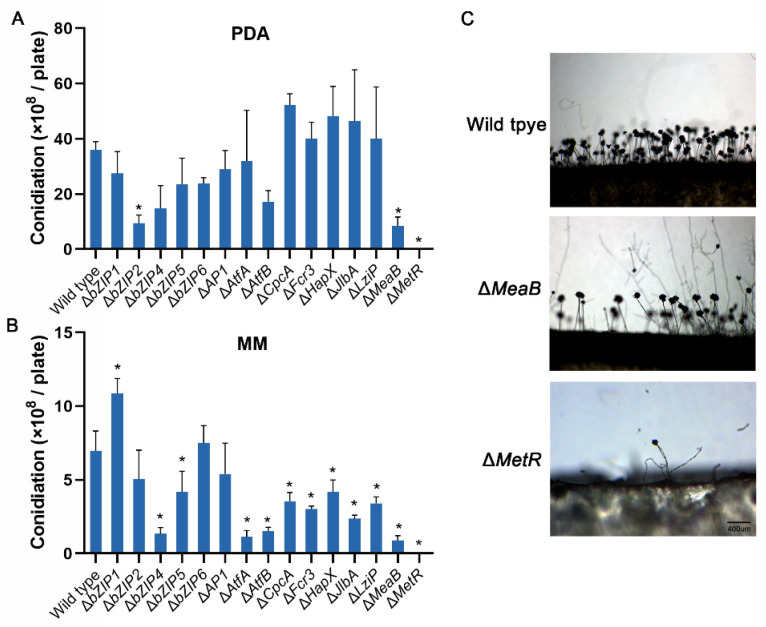
Analysis of conidial production of *Aspergillus flavus* strains. In total, 1 × 10^4^ spores were cultured on PDA (**A**) and MM (**B**) media for 7 days at 30 °C. The conidia produced per plate by the tested strains were numbered. Error bars represent the SD. * *p* < 0.05, significant difference from the wild-type group as estimated by a Dunnett test. (**C**) Conidiophores of the mutant strains Δ*MeaB* and Δ*MetR* on PDA. Bar = 400 μm. MM, minimal media; PDA, potato dextrose agar; SD, standard deviation.

The conidiation of HMN mutants of the 15 bZIP transcription factors was also studied. The Δ*bZIP2* and Δ*MeaB* mutants produced approximately 73.8% and 76.4% fewer conidia on PDA plates, respectively, compared with the wild-type fungus. In addition, the two mutants produced only sparse conidiophores. The Δ*MetR* mutant produced few conidiophores (Figure 5A,C).

Δ*MetR* could not grow on MM plates. The mutants of other 10 bZIP genes displayed defects in conidiation, and five of them, Δ*bZIP4*, Δ*AtfA*, Δ*AtfB*, Δ*JlbA*, and Δ*MeaB*, produced at least 70% fewer conidia compared with the wild-type. Δ*bZIP1* produced significantly more conidia than the wild-type fungus, with an increase of approximately 50% (Figure 5B).

### 3.7. bZIP Transcription Factors Involved in Sclerotial Development

**Figure 6:**
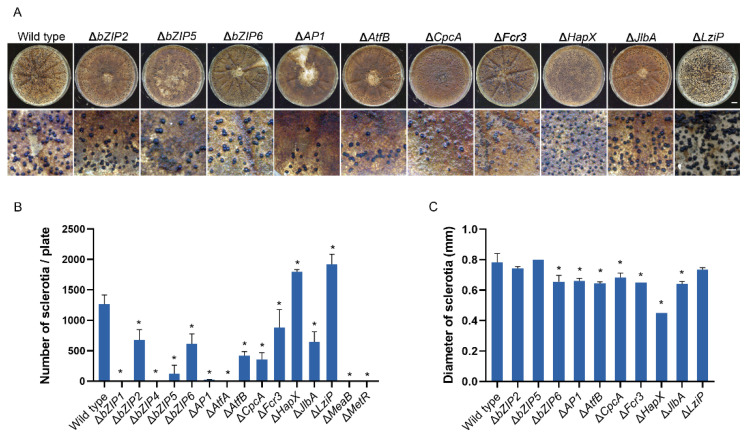
Analysis of the sclerotial production of *Aspergillus flavus* strains. (**A**) Colonies of mutants with sclerotia. In total, 1 × 10^4^ spores were cultured on 90 mm WKM plates in the dark for 10 days at 30 °C. Bar = 1 cm. (**B**) The number of sclerotia per plate. Bar = 2 mm. (**C**) Analyses of the size of sclerotia. Ten sclerotia were arranged in a row, and the length was measured and then converted into the diameter (mm) of a sclerotium. Error bars represent the SD. * *p* < 0.05, significant difference from the wild-type group as estimated using a Dunnett test. SD, standard deviation; WKM, Wickerham media.

The effects of deletion of the bZIP genes on sclerotial production were determined. When the HMN mutants were cultured on WKM in the dark for 10 days, the mutants of five bZIPs did not produce sclerotia, including Δ*bZIP1*, Δ*bZIP4*, Δ*AtfA*, Δ*MeaB* and Δ*MetR* (Figure S3). The mutants of seven bZIPs produced significantly fewer sclerotia compared with the wild-type (Figure 6A). The numbers of sclerotia of Δ*bZIP2*, Δ*bZIP6* and Δ*JlbA* were reduced by approximately 50%, Δ*AtfB* and Δ*CpcA* by approximately 70%, while Δ*bZIP5* and Δ*AP1* were reduced by at least 90%. However, Δ*HapX* and Δ*LziP* produced approximately 50% more sclerotia than the wild-type (Figure 6B). The size of sclerotia of these mutants was also determined. The mutants of *bZIP6*, *AP1*, *AtfB*, *CpcA*, *HapX* and *JlbA* genes all produced smaller sclerotia than the wild-type, and the sclerotia of Δ*HapX* were the smallest, decreased by 42.3% in diameter (Figure 6C, Table S3).

### 3.8. bZIP Transcription Factors Involved in Aflatoxin Production

**Figure 7:**
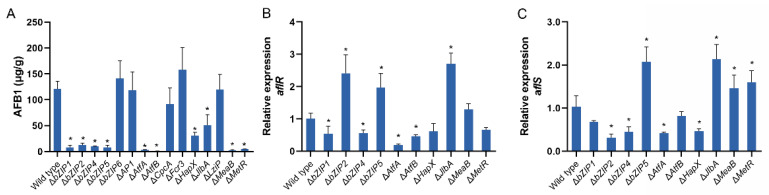
Analysis of the biosynthesis of aflatoxin by *Aspergillus flavus* strains. (**A**) Aflatoxin production by the *A. flavus* strains. Conidia of the indicated strains were inoculated on YES media. The production of AFB1 was determined using HPLC after 4 days of incubation at 28 °C. The relative levels of expression of *aflR* (**B**) and *aflS* (**C**) in mutants with reduced aflatoxin production. Error bars represent the SD. * *p* < 0.05, significant difference from the wild-type group as estimated by a Dunnett test. AFB1, aflatoxin B1; HPLC, high pressure liquid chromatography; SD, standard deviation; YES, yeast extract with supplements.

To study the effect of the deletion of bZIPs genes on the biosynthesis of aflatoxin, the production of AFB1 by the mutants of 15 bZIP genes was quantified by high performance liquid chromatography (HPLC). Our findings revealed that the mutants of 10 bZIP genes produced significantly lower amounts of AFB1 compared with the wild-type (121.5 µg/g), and eight produced <10% of AFB1, including Δ*bZIP1*, Δ*bZIP2*, Δ*bZIP4*, Δ*bZIP5*, Δ*AtfA*, Δ*AtfB*, Δ*MeaB* and Δ*MetR*. The production of AFB1 by Δ*HapX* and Δ*JlbA* was reduced at 74.6% and 57.9%, respectively (Figure 7A).

In these 10 mutants with reduced levels of AFB1, we also studied the expression of *aflR* and *aflS*, important positive regulators of the aflatoxin biosynthetic pathway (Figure 7B,C). The results showed that the expression of *aflR* in four mutants (Δ*bZIP1*, Δ*bZIP4*, Δ*AtfA* and Δ*AtfB*) was significantly downregulated at the same time. It is notable that the expression of *aflS* in the Δ*bZIP4* and Δ*AtfA* was also downregulated. In contrast, *aflS* in Δ*HapX* were downregulated, while there was no difference in the expression of *aflR* compared with the wild-type. However, *aflR* was significantly upregulated in three mutants (Δ*bZIP2*, Δ*bZIP5* and Δ*JlbA*). In particular, the level of expression of *aflS* in Δ*bZIP5* and Δ*JlbA* was also upregulated. In addition, only *aflS* was upregulated in two mutants (Δ*MeaB* and Δ*MetR*).

### 3.9. bZIP Transcription Factors Related to Oxidative Stress

**Figure 8:**
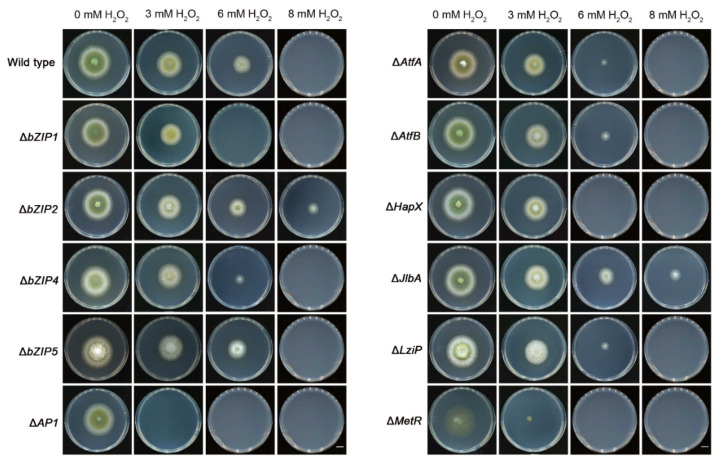
Effects of different concentrations of H_2_O_2_ on the growth of *Aspergillus flavus* strains. Conidia of the indicated strains were inoculated on PDA supplemented with H_2_O_2_ for 3 days at 30 °C. Bar = 1 cm. Growth inhibition rate (%) of 15 bZIPs mutants under H_2_O_2_ stress are shown in Table S3. H_2_O_2_, hydrogen peroxide; PDA, potato dextrose agar.

The sensitivities of 15 bZIPs mutants to oxidative stress were assayed by measuring their mycelial growth under 3, 6 and 8 mM hydrogen peroxide (H_2_O_2_). The wild-type fungus could not grow when treated with 8 mM H_2_O_2_. In comparison, Δ*AP1* was most sensitive to oxidative stress and could not grow under 3 mM H_2_O_2_. Seven bZIPs mutants were more sensitive to 6 mM H_2_O_2_. Δ*bZIP1*, Δ*HapX* and Δ*MetR* could not grow at all, while the growth of Δ*bZIP4*, Δ*AtfA*, Δ*AtfB*, and Δ*LziP* was significantly reduced under 6 mM H_2_O_2_. The mutants Δ*bZIP2* and Δ*JlbA* were significantly more tolerant to oxidative stress and could grow under 8 mM H_2_O_2_ (Figure 8, Table S3). In addition, Δ*MeaB* was only less sensitive to 3 mM H_2_O_2_ compared with the wild-type, although it was similarly affected by 6 and 8 mM H_2_O_2_ compared with the wild-type (Table S3).

### 3.10. bZIP Transcription Factors Related to Cell Wall, Osmotic, Acid and Alkali Stress

**Figure 9:**
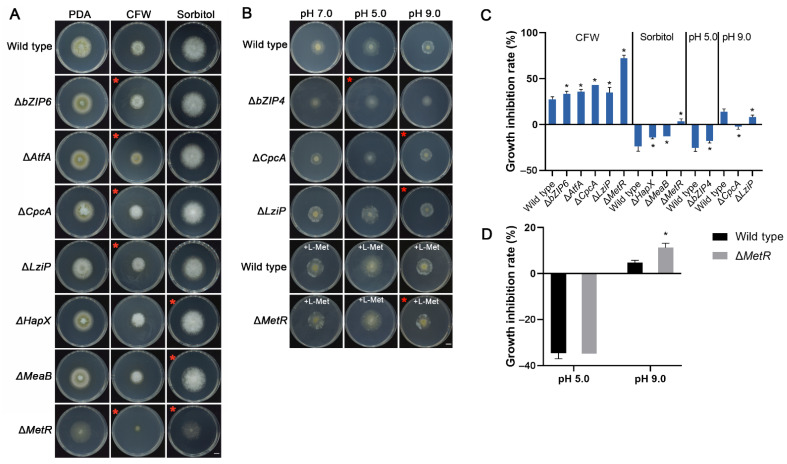
Effects of cell wall stress, osmotic stress, and acid and alkali stress on the growth of *Aspergillus flavus* strains. Mutants with significant differences are shown here. The rate of inhibition of the others is shown in Table S3. (**A**) Colonies of the mutants subjected to CFW and sorbitol stress. The mutant strains were inoculated on PDA supplemented with 400 µg/mL CFW for cell wall stress and 1.5 mM sorbitol for osmotic stress for 3 days at 30 °C. Bar = 1 cm. (**B**) Colonies of mutants under acid and alkali stress. bZIPs mutant strains that were inoculated on MM with pH 5.0 and pH 9.0 for 3 days at 30 °C, except that Δ*MetR* was inoculated on MM supplemented with 5 mM L-methionine (L-Met). pH 7.0 was used as the control. A red star indicates the mutant phenotype. Bar = 1 cm. (**C**) Growth inhibition rate of mutants under stresses. The rate of inhibition of mycelial growth was calculated by measuring the diameter of fungal colonies and normalized to the growth of control, respectively. (**D**) Growth inhibition rate of Δ*MetR*. The error bars represent the SD. * *p* < 0.05, significant difference from the wild-type group as estimated by a Dunnett test. CFW, Calcofluor white. MM, minimal media; PDA, potato dextrose agar; SD, standard deviation.

Various types of abiotic stress, such as cell wall, osmotic, acid and alkali stress, can affect the development and infection cycle of fungi. We studied the response of all bZIPs mutants to the four kinds of abiotic stress, including 400 µg/mL CFW, 1.5 mM sorbitol, pH 5.0 and pH 9.0. CFW is a cell wall stress compound. Five of the 15 bZIP mutants were more sensitive to this compound (Figure 9A). Notably, the growth of Δ*MetR* was inhibited by 2.6-fold compared with the wild-type (Figure 9D). Hypertonic pressure with 1.5 mM sorbitol unexpectedly promoted the growth of the wild-type and most mutants. The exceptions were Δ*HapX* and Δ*MeaB*, with growth that only increased by 14.1% and 12.8%, respectively, which was significantly lower than that of the wild-type (23.9%) (Table S3). In addition, only Δ*MetR* exhibited reduced growth under this osmotic stress (Figure 9B). Acidic conditions also promoted mycelial growth because pH 5.0 is suitable for the growth of *A. flavus*, and only Δ*bZIP4* differed significantly from the wild-type. Instead, most mutants grew poorly at pH 9.0 compared with pH 7.0, and only Δ*LziP* differed from the wild-type, while the growth of Δ*CpcA* increased by 2.4% at pH 9.0 (Figure 9C). In addition, Δ*MetR* could not grow on the MM media. Thus, MM media that had been supplemented with L-methionine were used for acid and alkali stress. The results showed that Δ*MetR* was more sensitive to alkali stress (Figure 9D).

### 3.11. bZIP Genes Required for Pathogenicity

**Figure 10:**
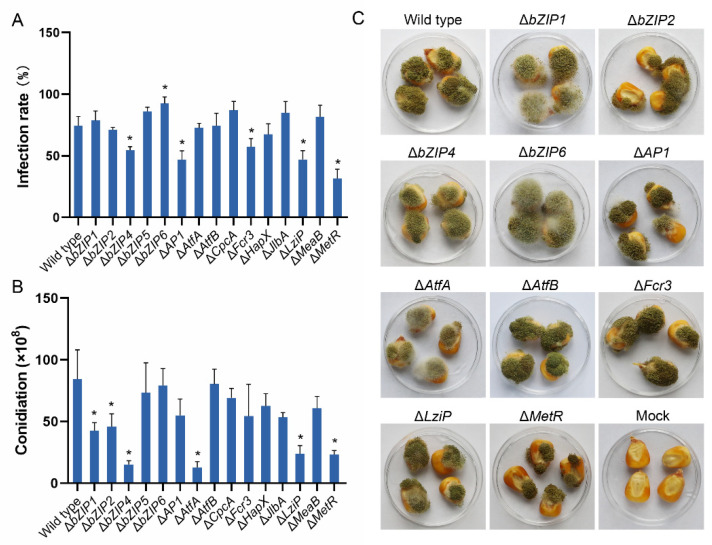
Pathogenicity assay of the *Aspergillus flavus* strains. Maize kernels were inoculated with a conidial suspension for 7 days. (**A**) Infection rate (%) of mutants. The rate of infection was calculated by taking the infected areas and dividing them by the surface areas of the kernels. (**B**) Conidial production of the mutants colonized on kernels. The conidia of mutants were harvested by washing the kernels with 0.01% Triton X-100 and then numbered. Error bars represent the SD. (**C**) Virulence assay of 10 mutants on maize kernels. The 10 mutants Δ*bZIP1*, Δ*bZIP2*, Δ*bZIP4*, Δ*bZIP6*, Δ*AP1*, Δ*AtfA*, Δ*AtfB*, Δ*Fcr3*, Δ*LziP* and Δ*MetR* differed significantly in infection rate or/and conidial production compared with the wild type. Bar = 0.5 cm. * *p* < 0.05, significant difference from the wild-type group as estimated by a Dunnett test. SD, standard deviation.

The virulence of 15 bZIPs null mutants was tested by inoculating maize kernels with conidial suspensions and evaluating the rate of infection and production of conidia. In this study, the infection rate was calculated from the area covered by hyphae and/or conidia divided by the kernel surface area. Three mutants, including Δ*bZIP4*, Δ*LziP*, and Δ*MetR*, were reduced in both their rate of infection and production of conidia (Figure 10A,B). Although Δ*AP1* and Δ*Fcr3* infected a smaller area than the wild-type, their production of conidia did not differ significantly from that on the maize kernels. In contrast, Δ*bZIP1*, Δ*bZIP2* and Δ*AtfA* had similar infection rates, but they produced fewer conidia. This was because Δ*bZIP1* and Δ*bZIP2* displayed more vigorous mycelial growth and dispersed conidia on kernels compared with the wild-type isolate that produced clustered and compact conidia (Figure 10C). In addition, the Δ*bZIP6* mutant had a higher rate of infection compared with the wild-type, but there was no difference in the production of conidia owing to the more vigorous growth of mycelia.

### 3.12. Hapx Is Important for A. flavus to Adapt to an Excess of Iron

**Figure 11:**
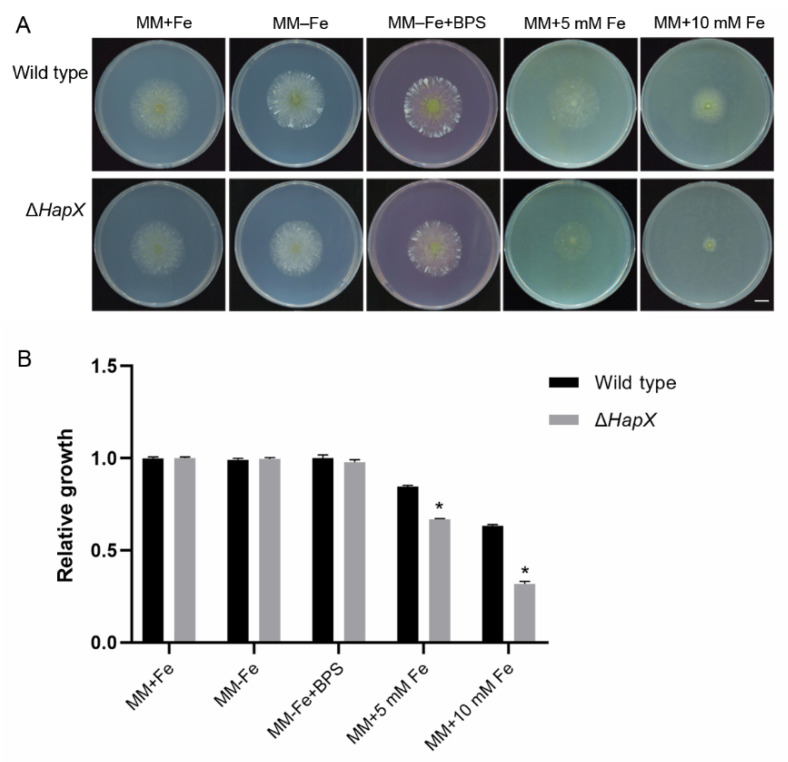
Deletion of HapX impairs fungal growth under conditions of high iron. (**A**) Colonies of the wild-type strain (NRRL 3357) and Δ*HapX* strain grown on MM+Fe (0.03 mM FeSO_4_), MM–Fe (no Fe), MM–Fe+BPS (0.2 mM BPS, but no Fe) plates, MM+5 mM Fe, and MM+10 mM Fe plates, respectively. The strains were cultured at 30 °C for 3 days. Bar = 1 cm. (**B**) Relative mycelial growth of the strains was obtained by measuring the diameter of fungal colonies and normalizing the data to the growth of wild-type and Δ*HapX* on MM+Fe, respectively. Error bars represent the SD. * *p* < 0.05, significant difference from the wild-type group estimated by a Dunnett test. BPS, bathophenanthroline disulfonate. MM, minimal media; SD, standard deviation.

Since HapX was identified as important to sustain iron homeostasis in *A. nidulans* [58] and other fungal pathogens [30,32,34], we investigated whether HapX has a similar role in *A. flavus*. To control the level of iron, MM that lacked FeSO_4_ (MM–Fe) was used as the iron deficiency condition. The addition of 0.2 mM of the iron chelator bathophenanthroline disulfonate (BPS) and 0.03 mM FeSO_4_ to MM–Fe were used as iron starvation and iron sufficient conditions, respectively. MM was supplemented with 5 or 10 mM FeSO_4_ to examine the parameters under conditions of high iron. Growth assays were performed with 1 μL of conidial suspension (10^6^ spores/mL) inoculated on solid media and incubated at 30 °C for three days. Growth analyses revealed that the amount of radial growth between the wild-type and mutant was similar between MM–Fe or MM–Fe+BPS and MM+Fe. The radial growth of Δ*HapX* and the wild-type were all reduced following treatment with high amounts of iron (Figure 11). Furthermore, the relative growth of Δ*HapX* was dramatically lower than that of the wild-type, which suggested that the HapX deletion mutant was more sensitive to high iron conditions compared with the wild-type.

### 3.13. MetR and Methionine Biosynthesis Is Important for the Development of A. flavus

**Figure 12:**
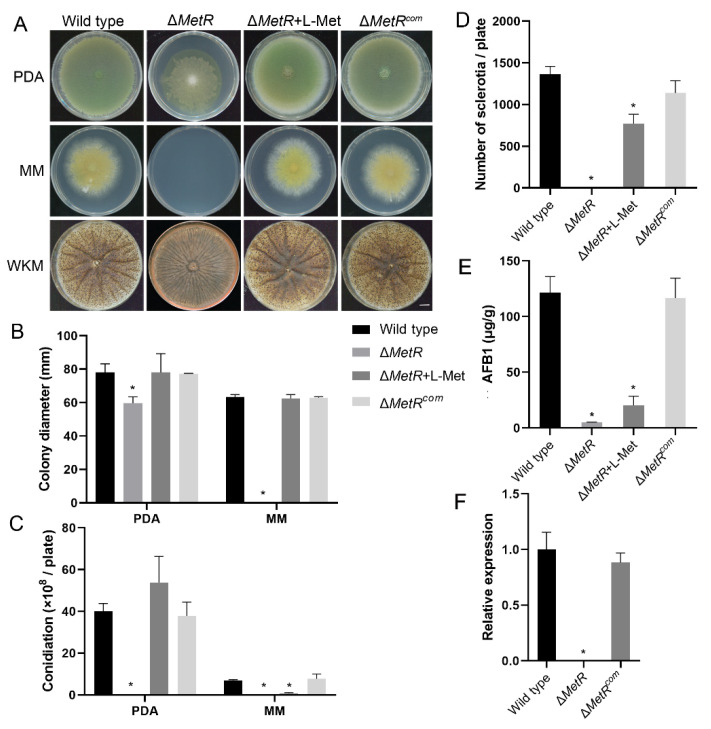
Phenotypic analysis of the Δ*MetR* and Δ*MetR^com^* strains. (**A**) Colonies of the wild-type strain, Δ*MetR* mutant and complemented strain (Δ*MetR^com^*) on different media. Each strain was inoculated on PDA and MM for 7 days at 30 °C and on WKM medium for 10 days at 30 °C in dark. In addition, Δ*MetR* was inoculated on three other types of media supplemented with 5 mM L-methionine (L-Met). Bar = 1 cm. (**B**) Colony diameter of each strain. (**C**) Conidial production of each strain. (**D**) Sclerotial production of each strain. Bar = 5 mm. (**E**) Aflatoxin B1 production measured per μg/g of mycelia. (**F**) A relative expression assay of the *MetR* gene in Δ*MetR^com^* using quantitative PCR. Error bars represent the SD. * *p* < 0.05, significant difference from the wild-type group estimated by a Dunnett test. MM, minimal media; PDA, potato dextrose agar; SD, standard deviation; WKM, Wickerham media.

In addition, the MetR mutants are tight auxotrophs that require methionine for fungal growth. In our study, the deletion of MetR significantly affected its mycelial growth, conidiation, sclerotial formation and aflatoxin biosynthesis. Methionine was added to the culture to determine whether these phenotypes were owing to a defect of methionine biosynthesis in Δ*MetR*. This showed that Δ*MetR* could restore normal mycelial growth to both PDA and MM cultures in which L-methionine (L-Met) was added (Figure 12A,B). The mutant could also restore normal conidiation in which L-Met was added to PDA. However, Δ*MetR* produced fewer conidia when L-Met was added to MM and only produced approximately 12% compared with the wild-type (Figure 12C). MM is a basic medium for fungal growth and contains fewer nutrients than PDA. Our results suggest that MetR may regulate other metabolic pathways that affect conidiation other than methionine biosynthesis. We studied the effect of methionine supplementation on the production of sclerotia and AFB1 in culture in more detail. This showed that the addition of methionine to the mutants could partially restore approximately 56% and 16.7% of the wild-type, respectively (Figure 12D,E). These results suggest that MetR could be involved in the regulation of production of sclerotia and aflatoxin production in a pathway other than methionine biosynthesis.

To confirm that the defects of the mutant were caused by the knockout of the MetR transcription factor, the mutant Δ*MetR* was complemented with its native copy from the wild-type isolate NRRL 3357. The phenotypic analyses showed that Δ*MetR^com^* recovered from the defects in mycelial growth, conidial and sclerotial development, and the production of aflatoxin when compared with Δ*MetR* and wild-type (Figure 12A–E). These results were also reconfirmed at the transcriptional level (Figure 12F).

## 4. Discussion

The construction of mutants based on homologous recombination has been a powerful tool for functional genomic research in some fungi, such as the yeasts *S. cerevisiae* and *Schizosaccharomyces pombe* and the filamentous fungi *N. crassa* and *M. oryzae*. However, in *A. flavus*, protoplast transformation has been the primary system for gene-deletion analysis to date, which is laborious, highly inefficient, and difficult to apply to high-throughput gene function analyses. Alternatively, the mycelia and conidia of *A. flavus* are multinucleate, which is another obstacle for gene-deletion assays. In this study, we developed a double-fluorescence gene knockout strategy based on a previously established ATMT system. This strategy is available to delete large numbers of genes by enabling the construction of highly efficient gene knockouts that result in a reliable and labor-saving screening methods for transformants. During this procedure, the gene-deletion cassettes were generated by in vivo recombination in yeast using a yeast–*Escherichia*–*Agrobacterium* shuttle vector pKO, which could also be replicated in *E. coli* and *Agrobacterium* cells. A similar vector construction, pKO1B, was first reported by Jianping Lu, which was successfully used for the deletion of genes for 104 Zn2Cys6 and 47 Cys2-His2 transcription factors in *M. oryzae* [56,59]. The pKO1B vector only used GFP fluorescence as a negative marker to eliminate ectopic insertion transformants. The null mutants with no fluorescence could be further distinguished from the wild-type through negative screening double PCR for the target and *β-tubulin* genes. In this study, the pKO vector also used GFP fluorescence as a negative marker to eliminate ectopic insertion transformants. The targeted gene (x)-deletion cassettes in the pKO-x vector that contained RFPs fused with the resistance gene *ble* and were used as a positive marker for putative null mutants to exclude the wild-type. However, it is more complex to screen for null mutants in this fungus because the conidia of *A. flavus*, the receptor for ATMT transformation in our procedure, are multinucleate [60]. It has been estimated that approximately 70% of the cells have two nuclei, and 5% had even more nuclei in the conidia of *A. flavus* NRRL 3357 [61]. Although we tried to collect uninucleate conidia by filtering them through a membrane, there was still a small number of multinucleate conidia, which resulted in a few putative null mutants that harbored heterogeneous nuclei (HTN) in which a wild-type nucleus and recombinational nucleus coexisted in the same strain. The HTN would interfere with the phenotypic identification of the mutants and functional analysis of the genes. Similar to the null mutants with homogeneous nuclei (HMN), the mutants with HTN also emit red fluorescence under UV. However, we could identify the HMN mutants and exclude the HTN mutants through negative double PCR of the target and *β-tubulin* genes. In addition, the null mutants with HMN could be verified by positive PCR of the gene-deletion cassettes. In summary, the double-fluorescence knockout construction in this procedure provides a more convenient strategy for the functional analysis of gene deletions in fungi than the mono-fluorescence one. RFP fluorescence was used as a positive marker to eliminate wild-type stains. For fungi with uninucleate cells, the transformants that only fluoresce red can be confirmed as null mutants. This advantage eliminates laborious work and makes it easy to screen null mutants, particularly for fungi with multinucleate cells.

In this study, we identified 17 bZIP transcription factors in *A. flavus* and finally generated 15 bZIP TF gene-deleted null mutants out of 17 selected bZIP genes. The phenotypes of 15 bZIP TF null mutants indicated that these bZIP transcription factors participated in many critical cellular processes in this fungus, such as mycelia growth, conidiogenesis, sclerotial development, aflatoxin biosynthesis and defense against oxidative, cell wall, osmotic and acid and alkali stresses and pathogenicity in *A. flavus*. The TF *MetR* was simultaneously involved in nine tested biological process, two genes (*AtfA* and *bZIP4*) were involved in seven processes, three TF genes (*bZIP1*, *bZIP2* and *LziP*) were involved in six processes, five genes (*AtfB*,*CpcA*, *HapX*, *JlbA* and *MeaB*) were involved in five processes, and four genes (*bZIP5*, *bZIP6*, *Ap1* and *Fcr3*) were involved in three processes (Table 1 and Table S3). Another two bZIP TF genes, *bZIP3* and *HacA*, were only obtained in HTN mutants by two rounds of transformations with MM selection media and one round of transformation with PDA selection media. HacA, an ortholog of Hac1 in *S. cerevisiae* [62], is a master transcriptional regulator of the unfolded protein response (UPR) that originates in the endoplasmic reticulum (ER) and coordinates protein folding, secretion, phospholipid biosynthesis and protein degradation [25,63]. The deletion of *HacA* did not seriously affect fungal growth in such species as *A. fumigatus* [64], *A. oryzae* [27], and *Trichophyton rubrum* [28]. The unavailability of HMN in the *HacA* mutants in our study suggested that this gene is essential for fungal growth in *A. flavus*.

Among the 15 bZIP TFs, *MeaB*, *AP1* and *Fcr3* (*AflRsmA* ortholog) have been identified in *A. flavus* and were also included in our knockout assay. A previous study showed that the deletion of *MeaB* did not affect conidiation, the production of sclerotia and AFB1, and pathogenicity [40]. The mutant Δ*MeaB* displayed a statistically significant reduction in conidiogenesis, and the production of AFB1 and did not produce any sclerotia, although it remained pathogenic. *AP1* has been reported to play a key role in the regulation of oxidative stress and aflatoxin production in *A. flavus* [50]. In contrast, the deletion of *AP1* did not significantly affect the production of aflatoxin. In addition, we proved that *AP1* is involved in the formation of sclerotia, which has not been reported to the best of our knowledge. All the divergency in mutant phenotypes could owe to the differences in wild-type isolates or experimental conditions. *AflRsmA* is another bZIP TF from *A. flavus* that has recently been reported. It is highly homologous with *Fcr3* in this study. The *AflRsmA* gene in *A. flavus* was found from the start codon of AFLA_133570 to the stop codon of AFLA_133560 and consisted of 1070 bp with two introns (47 and 102 bp). It encodes a 305 aa protein [51]. The AFLA_133560 gene is annotated as *Fcr3* in the NCBI, which indicates that *Fcr3* is one part of the *AflRsmA* gene structure. Nevertheless, we deleted *Fcr3* based on the NCBI data and showed that the Δ*Fcr3* mutant had attenuated conidiation, sclerotia and virulence, which was consistent with Δ*AflRsmA*.

*AtfA* and *AtfB* have been confirmed to be involved in conidial development, stress responses, and secondary metabolism in other species of *Aspergillus*, such as *A. nidulans* [65], *A. fumigatus* [66], and *A. parasiticus* [67,68,69]. This study revealed that the deletion of these two genes led to attenuated conidiation, more sensitivity to H_2_O_2_ stress and a decrease in AFB1. Furthermore, sclerotial development and virulence were also affected in Δ*AtfA* and Δ*AtfB*. Impressively, Δ*AtfA* did not produce sclerotia and produced the fewest number of conidia on maize kernels.

*CpcA*, a homolog of *Gcn4* in *S. cerevisiae* and *Cpc1* in *N. crassa* [70,71], has been reported to act as a novel regulator of the anabolism of amino acids in filamentous fungi, such as *A. nidulans* [72], *A. fumigatus* [24], and *A. niger* [73]. The disruption of *CpcA* resulted in sensitivity to amino acid deprivation generated by the histidine analog 3-aminotriazole (3AT), which is an inhibitor of amino acid biosynthesis. *JlbA*, another *jun*-like bZIP gene, has also been found to have a similar function in amino acid biosynthesis [23,74]. In our study, the wild-type itself was sensitive to 3AT, and there was almost no growth in the culture supplemented with 1 mM of 3AT. When the strains were grown with <1 mM 3AT, there was no significant difference in mycelial growth and conidiation between Δ*CpcA*, Δ*JlbA* and the wild-type. However, Δ*JlbA* mutant strains produced less aflatoxin and had an increased resistance to oxidative stress. In addition, the production of sclerotia by the two mutants, Δ*CpcA* and Δ*JlbA*, was dramatically reduced compared with the wild-type.

In this study, we also demonstrated that HapX was not only an important regulator of fungal conidiation, sclerotial development, aflatoxin biosynthesis and oxidative stress, but it was also involved in iron metabolism in *A. flavus*. Δ*HapX* produced fewer conidia and lower amounts of AFB1, and it was more sensitive to H_2_O_2_ stress. Impressively, the sclerotial production of the mutant increased, but the sizes of the sclerotia were 0.45 mm. This was a dramatic decrease in size when compared with 0.78 mm for the wild-type. In addition, previous studies have revealed that the HapX transcription factor is a major regulator of iron homeostasis, enabling adaptation to both low and excessive amounts of iron [30,34,58]. However, we demonstrated that the *HapX* deletion mutant of *A. flavus* only showed an increased sensitivity to excessive amounts of iron and not to iron deficiency (MM–Fe) and iron starvation (MM+BPS) conditions. Notably, Δ*HapX* displayed a strong growth defect compared with the wild-type in the presence of 10 mM Fe, which suggests that the regulatory mechanism of HapX transcription factor may differ in various strains.

The MetR transcription factor is a positive regulator of sulfur metabolism in *A. nidulans* [39]. It plays an important role in inorganic sulfur acquisition and is functionally similar to Met4 in *S. cerevisiae* [75] and Cys3 in *N. crassa* [76]. As expected, the deletion of *MetR* in *A. flavus* resulted in methionine auxotrophy in MM cultures that only contained sulfate. Although the growth of Δ*MetR* can be restored by supplementation with exogenous methionine, this was not the case with conidiogenesis on the MM media. In *A. fumigatus* [36] and *M. oryzae* [13], the *MetR* deletion mutant exhibited similar responses. Except for fungal growth and conidiation, *MetR* has also been found to be involved in oxidative stress and virulence in *Alternaria alternata* [37]. In *Serratia marcescens*, a Gram-negative bacterium, *MetR* was also found to be related to tolerance to H_2_O_2_ [77]. In this study, Δ*MetR* displayed defects not only in resistance to oxidative stress and virulence but also in sclerotial development and aflatoxin biosynthesis. Notably, the defects in sclerotia and AFB1 production were not fully recovered by exogenous methionine, which indicated that *MetR* may regulate the asexual development and aflatoxin biosynthesis beyond the biosynthetic pathway for methionine in this fungus. In addition, we also found that Δ*MetR* was more sensitive to cell wall, osmotic and alkali stresses. When the media were supplemented with methionine, Δ*MetR* was restored to its normal phenotype under cell wall and osmotic stress but not under alkali stress. Δ*MetR^com^* recovered under all three types of stress (unpublished data). This suggests that *MetR* regulates the resistance to alkali stress and is not related to the metabolism of methionine in this fungus.

In the *A. flavus* genomic database, the gene AFLA_083100 was annotated as *LziP*, which has been characterized in humans and mice, and found that the leucine zipper of *LZIP* was slightly longer and different from other members of the bZIPs family [78,79]. However, there was no characterized ortholog in plants and fungi until now. Our results indicated that *LziP* of *A. flavus* is involved in conidiation, sclerotial development, oxidative stress and pathogenicity. In particular, the deletion of *LziP* led to an increase in the production of sclerotia, which were approximately 1.5-fold higher than those produced by the wild-type. However, the size of sclerotia did not differ from those of the wild-type.

The remaining unannotated six bZIPs (*bZIP1*~*bZIP6*) in *A. flavus* were first studied here. Except for *bZIP3* without the HMN mutant, the phenotypes of the other five bZIP mutants were all analyzed. The deletion of *bZIP6* only affected sclerotial development in this study, which suggested that it could function as a local regulator of fungal development. Other bZIPs, including *bZIP1*, *bZIP2*, *bZIP4* and *bZIP5*, were all involved in conidiation, sclerotial development, aflatoxin biosynthesis and oxidative stress. Notably, their mutants all had a dramatic decrease in the amount of aflatoxin produced. Δ*bZIP1* and Δ*bZIP4* did not produce sclerotia; Δ*bZIP2* displayed an increased resistance to oxidative stress, and Δ*bZIP4* had reduced virulence. These results suggest that these bZIPs may be upstream regulators or located on critical nodes of regulatory network. They clearly play an important role in multiple biological processes in *A. flavus.*

*A. flavus* is the dominant fungus that produces aflatoxins. It has been confirmed that the genes for aflatoxin biosynthesis are located in the 70 kb gene cluster of this fungus. The expression of genes in the cluster is positively regulated by *aflR* and *aflS* [80]. However, the exact regulatory mechanism for aflatoxin biosynthesis has not yet been completely elucidated. For example, the deletion of the regulators *NsdC* and *NsdD* resulted in a decline in aflatoxin production, but the expression of *aflR* was normal [81]. In our study, 10 bZIP gene mutants produced significantly lower amounts of AFB1. Among them, four bZIPs (*bZIP1*, *bZIP4*, *AtfA* and *AtfB*) could be AflR/AflS-dependent regulatory factors. However, *aflR*/*aflS* were normal or upregulated in six other bZIP mutants, which suggested that these genes may regulate the biosynthesis of aflatoxin in *A. flavus* in an unknown manner. We speculated that there might be two reasons: (1) the post-translational regulation of phosphorylation of AflR [82], which is quicker than the expression of AflR; (2) multiple function of AflR, which is involved in the fungal growth and development in addition to aflatoxin biosynthesis [5].

It has been reported that the regulation of secondary metabolism in filamentous fungi is closely linked with the cellular response to oxidative stress [83,84,85]. In *Aspergillus*, bZIP transcription factors, such as AP1, AtfA, and AtfB, have been confirmed to contribute to the co-regulation of aflatoxin biosynthesis and oxidative stress. Our results showed that, among the 10 bZIPs in which the production of aflatoxin was affected, all except for Δ*bZIP5* could also respond to oxidative stress. Δ*bZIP2* and Δ*JlbA* were more resistant to H_2_O_2_ compared with the other seven bZIPs mutants that were more sensitive to H_2_O_2_ stress. This suggests that these bZIP TFs might co-regulate the biosynthesis of aflatoxin and the response to oxidative stress in different manners. In addition, the mutants deleted in *AP1* and *LziP* were more sensitive to H_2_O_2_, but this did not significantly affect their AFB1 production. This suggests that the response to oxidative stress may not arbitrarily affect the biosynthesis of aflatoxin.

## 5. Conclusions

In this study, 15 bZIP transcription factors in *A. flavus* were characterized by a high-throughput knockout strategy based on an ATMT genetic transformation system. Gene knockout construction by yeast recombinational cloning and the screening of null mutants by double fluorescence provide an efficient way to construct gene-deleted mutants for this multinucleate strain. We generated 15 bZIPs gene-deleted null mutants with homogeneous nuclei. These bZIP transcription factors function as important regulators that are involved in many cellular processes, such as mycelial growth, conidiogenesis, sclerotial development, aflatoxin biosynthesis, nutrient utilization, defenses against oxidative, cell wall, osmotic and acid and alkali stresses, and pathogenicity in *A. flavus*. These studies will help us to further investigate the regulatory mechanism of bZIP TFs in *A. flavus* and uncover respective sites in the regulatory network.

## Figures and Tables

**Table 1:** Phenotypic summary of 15 bZIP transcription factor gene-deleted mutants.

Mutant	Growth	Conidiation	Sclerotia	Aflatoxin	Oxidative Stress	Cell Wall Stress	Osmotic Stress	Acid and Alkali Stress	Pathogenicity
Δ*bZIP1*	Reduced ^1^	Increased ^2^	None	Reduced	Affected ↓	No	No	No	Affected ^6^
Δ*bZIP2*	Reduced ^1^	Reduced ^1^	Reduced	Reduced	Affected ↑	No	No	No	Affected ^6^
Δ*bZIP4*	Reduced ^2^	Reduced ^1^	None	Reduced	Affected ↓	No	No	Affected ^3^	Affected ^5,6^
Δ*bZIP5*	Normal	Reduced ^1^	Reduced	Reduced	No	No	No	No	No
Δ*bZIP6*	Normal	Normal	Reduced	Normal	No	Affected	No	No	Affected ^5^
Δ*AP1*	Normal	Normal	Reduced	Normal	Affected ↓	No	No	No	Affected ^5^
Δ*AtfA*	Reduced ^2^	Reduced ^1^	None	Reduced	Affected ↓	Affected	No	No	Affected ^6^
Δ*AtfB*	Reduced ^2^	Reduced ^1^	Reduced	Reduced	Affected ↓	No	No	No	No
Δ*CpcA*	Reduced ^2^	Reduced ^1^	Reduced	Normal	No	Affected	No	Affected ^4^	No
Δ*Fcr3*	Normal	Reduced ^1^	Reduced	Normal	No	No	No	No	Affected ^5^
Δ*HapX*	Normal	Reduced ^1^	Increased	Reduced	Affected ↓	No	Affected	No	No
Δ*JlbA*	Reduced ^2^	Reduced ^1^	Reduced	Reduced	Affected ↑	No	No	No	No
Δ*LziP*	Normal	Reduced ^1^	Increased	Normal	Affected ↓	Affected	No	Affected ^4^	Affected ^5,6^
Δ*MeaB*	Normal	Reduced ^1,2^	None	Reduced	Affected ↓	No	Affected	No	No
Δ*MetR*	Reduced ^1,2^	Reduced ^1,2^	None	Reduced	Affected ↓	Affected	Affected	Affected ^4^	Affected ^5,6^

Note: The phenotypes of the mutants in colony growth, conidiation, sclerotia and aflatoxin production, response to stress, and pathogenicity were compared with the wild-type strain NRRL 3357. ^1^, strains cultured on PDA plates; ^2^, strains cultured on MM plates; ^3^, affected by acid stress; ^4^, affected by alkali stress; ^5^, infection rate of maize kernels by mutants; ^6^, conidial production of mutants on infected kernels; ↑, increased resistance to H_2_O_2_; ↓, decreased resistance to H_2_O_2_. H_2_O_2_, hydrogen peroxide; None, no sclerotia; No, unaffected.

## Data Availability

Data are contained within the article or Supplementary Material.

## Supplementary Materials

The following supporting information can be downloaded at: https://www.mdpi.com/article/10.3390/jof8040356/s1, Figure S1: Identification of gene-deleted mutants by PCR and Southern blot. (A) Knockout event of HMN mutants confirmed by PCR. a, bands for the target genes; b, bands for *β-tubulin*; c, bands for recombinational DNA fragments. Δ, the mutants; WT, wild-type. (B) HMN mutants of two bZIP genes confirmed by Southern blot. HMN, homogeneous nuclei; Figure S2: Colony morphology of 15 bZIP mutants on PDA (A) and MM (B) media. Each strain included front and back photos. MM, minimal media; PDA, potato dextrose agar; Table S1: The primers used in this study. (A) bZIP genes and primers used to amplify flanking fragments of the targeted genes in knockout. (B) Primers used to amplify the targeted genes in negative PCR. (C) Primers used to amplify a unique recombinational DNA fragment of null mutants by positive screening PCR. (D) Other primers used in this study; Table S2: Statistics of *Aspergillus flavus* transformants and knockout rate; Table S3: Phenotypic analyses of the HMN mutants of 15 bZIP transcription factor genes. HMN, homogeneous nuclei.
