# Supplementary material for: Systematic Characterization of bZIP Transcription Factors Required for Development and Aflatoxin Generation by High-Throughput Gene Knockout in Aspergillus flavus
Source: J Fungi (Basel). 2022 Mar 30;8(4):356. doi: 10.3390/jof8040356 (PMC9031554; doi:10.3390/jof8040356)
Supplement: Supplementary file 1 [file jof-08-00356-s001.zip › jof-1607872-supplementary Figure.pdf]

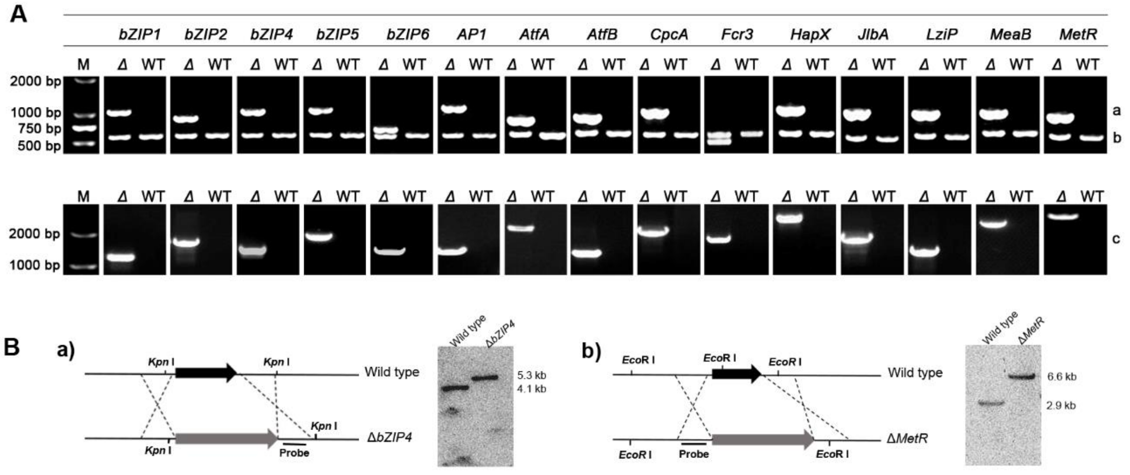

Figure S1: Identification of gene-deleted mutants by PCR and Southern blot. (A) Knockout event of HMN mutants confirmed by PCR. a, bands for the target genes; b, bands for *β-tubulin*; c, bands for recombinational DNA fragments. Δ, the mutants; WT, wild-type. (B) HMN mutants of two bZIP genes confirmed by Southern blot. HMN, homogeneous nuclei.

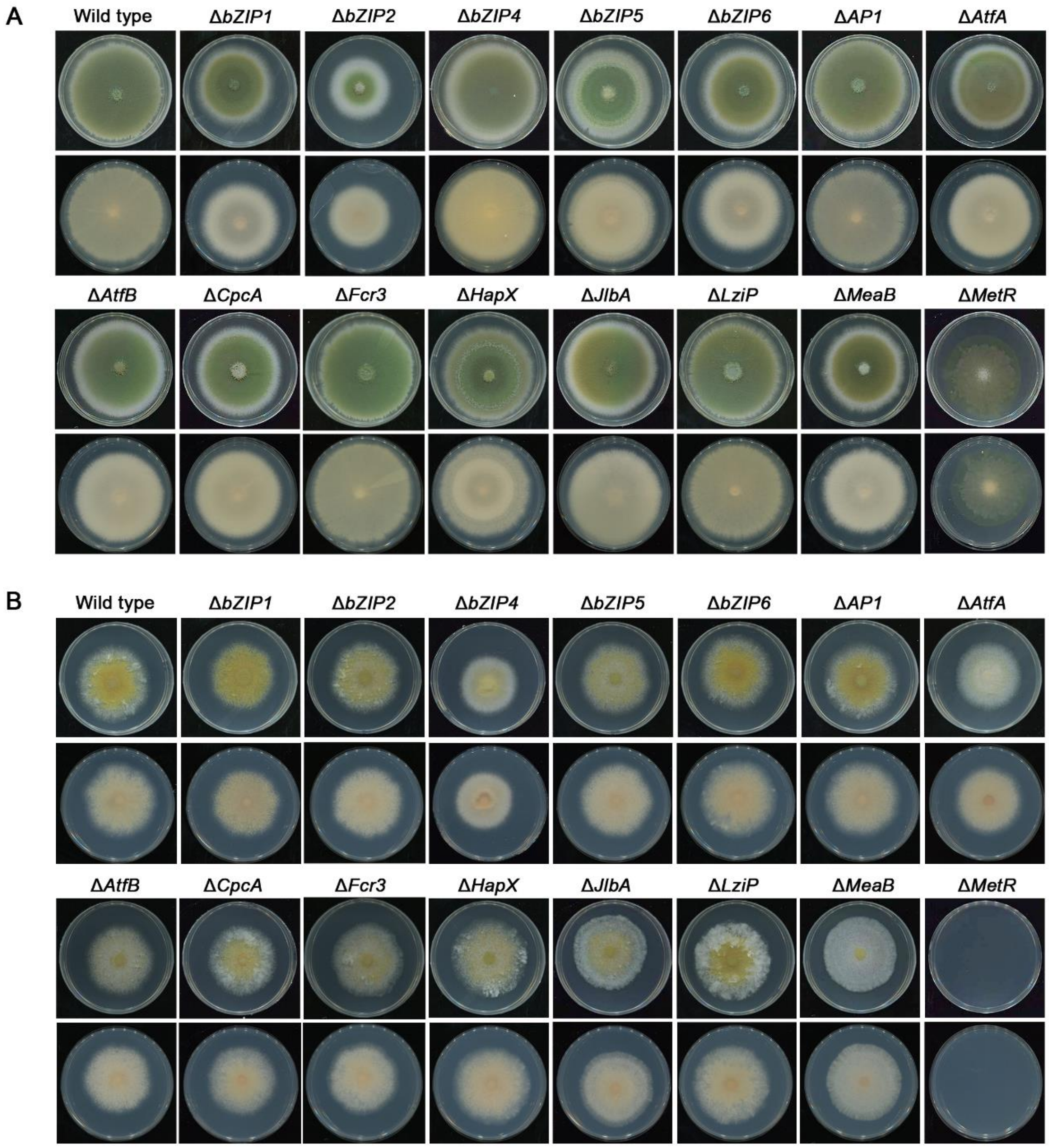

Figure S2: Colony morphology of 15 bZIP mutants on PDA (A) and MM (B) media. Each strain included front and back photos. MM, minimal media; PDA, potato dextrose agar.
